# Intestinal Alkaline Phosphatase: A Review of This Enzyme Role in the Intestinal Barrier Function

**DOI:** 10.3390/microorganisms10040746

**Published:** 2022-03-30

**Authors:** Gilberto Maia Santos, Shámila Ismael, Juliana Morais, João R. Araújo, Ana Faria, Conceição Calhau, Cláudia Marques

**Affiliations:** 1 Nutrition and Metabolism, NOVA Medical School, Faculdade de Ciências Médicas, Universidade NOVA de Lisboa, 1169-056 Lisboa, Portugal; gilberto.santos@nms.unl.pt (G.M.S.); shamila.ismael@nms.unl.pt (S.I.); juliana.morais@nms.unl.pt (J.M.); joaoricardo.araujo@nms.unl.pt (J.R.A.); ana.faria@nms.unl.pt (A.F.); ccalhau@nms.unl.pt (C.C.); 2 CINTESIS—Center for Health Technology Services Research, NOVA Medical School, Faculdade de Ciências Médicas, Universidade NOVA de Lisboa, 1169-056 Lisboa, Portugal; 3 CHRC—Comprehensive Health Research Centre, CEDOC—Chronic Diseases Research Center, NOVA Medical School, Faculdade de Ciências Médicas, Universidade NOVA de Lisboa, 1169-056 Lisboa, Portugal; 4 Unidade Universitária Lifestyle Medicine José de Mello Saúde by NOVA Medical School, 1169-056 Lisboa, Portugal

**Keywords:** inflammatory bowel disease, intestinal alkaline phosphatase, intestinal barrier function, low-grade chronic inflammation, metabolic dysfunction, necrotizing enterocolitis, obesity

## Abstract

Intestinal alkaline phosphatase (IALP) has recently assumed a special relevance, being the subject of study in the prevention and treatment of certain diseases related to leaky gut. This brush border enzyme (ecto-enzyme) plays an important role in the maintenance of intestinal microbial homeostasis and intestinal barrier function through its ability to dephosphorylate lipopolysaccharide (LPS). This review addresses how IALP and intestinal barrier dysfunction may be implicated in the pathophysiology of specific diseases such as inflammatory bowel disease, necrotizing enterocolitis, and metabolic syndrome. The use of IALP as a possible biomarker to assess intestinal barrier function and strategies to modulate IALP activity are also discussed.

## 1. Introduction 

Alkaline phosphatase (ALP), first described in 1907 by Suzuki et al. [1], is a glycoprotein bound to plasma membranes [2,3] that hydrolyzes several monophosphate esters optimally at an alkaline pH with the release of inorganic phosphates [4,5,6,7,8]. 

Although structurally and functionally distinct from protein phosphatases, some studies have shown that ALP is also capable of dephosphorylating proteins [9,10,11]. Protein phosphorylation/dephosphorylation balance plays an important role in the regulation of several cellular functions, such as proliferation and differentiation. Thus, it is possible that ALP may also be involved in the regulation of those functions as well [10,12,13].

Human ALP is divided into four isoenzymes depending on the tissue where it is expressed: placental ALP; germ cell ALP; liver, bone, or kidney ALP (tissue non-specific ALP); and intestinal ALP (IALP) [3]. 

IALP is an ectoenzyme usually expressed in intestinal epithelial cells (enterocytes), and their levels vary along the longitudinal axis of the intestine [10,14,15]. This brush border enzyme is involved in fatty acid absorption and plays an important role in the maintenance of intestinal microbial homeostasis and intestinal barrier function through its ability to dephosphorylate lipopolysaccharide (LPS). Tissue-non-specific ALP may also be present in the intestine, having the same functions as IALP [16]. IALP is also involved in the regulation of the intestinal surface pH and in the control of the composition, function, and anatomical location of intestinal microbiota constituents [8]. 

This review focuses on intestinal alkaline phosphatase (IALP) and on its role in the maintenance of the intestinal barrier function. In addition, this review addresses how IALP and intestinal barrier dysfunction may be implicated in the pathophysiology of some specific diseases such as inflammatory bowel disease, necrotizing enterocolitis, and metabolic syndrome. Gut microbiota dysbiosis and increased intestinal permeability have been recently pointed out as mechanisms involved in the etiology of these diseases, and in this context, IALP may assume a special relevance not only as a biomarker but also as a preventive and therapeutic strategy. The use of IALP as a possible biomarker to assess intestinal barrier function and strategies to modulate IALP activity is also discussed.

## 2. IALP and Intestinal Barrier Function

Trillions of generally harmless bacteria inhabit the surface of the host’s intestinal epithelium [17]. However, these bacteria can cause infection and septic shock and can be a threat if a protective intestinal barrier does not exist [17]. The intestinal barrier is what protects the host from an invasion of microorganisms, maintaining homeostasis and preventing infections [17,18]. The first lines of defense essential to maintaining the intestinal barrier are the mucus layer and antimicrobial peptides (AMPs) [18]. Tight junctions between intestinal epithelial cells and the lamina propria, where innate immune cells reside, are also essential for the integrity of the intestinal barrier [17,18].

**Figure 1:**
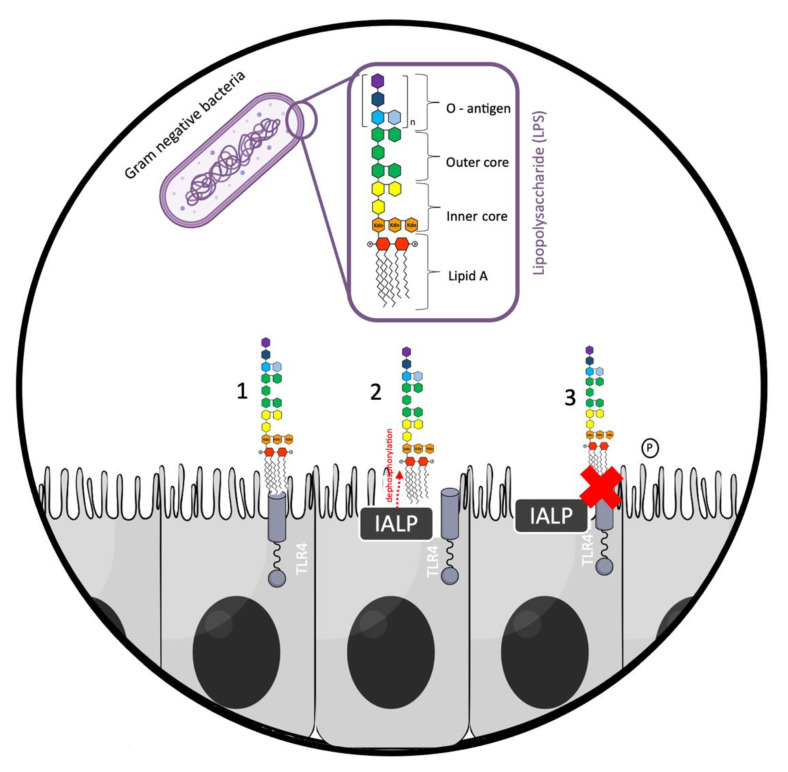
Detoxification of Gram-negative bacterial lipopolysaccharide (LPS) by intestinal alkaline phosphatase (IALP). 1—Absence of intestinal alkaline phosphatase: LPS binds to Toll-like receptor 4 (TLR4); 2—presence of IALP: lipid A phosphate group from LPS is dephosphorylated; 3—LPS dephosphorylation: no binding of LPS to TLR4 receptor, preventing the triggering of an inflammatory cascade. The red cross in the figure means no binding of LPS to TLR4.

Present in the membrane of intestinal cells, pattern recognition receptors (PRRs), such as Toll-like receptors (TLRs), are innate immune receptors that can detect highly conserved bacterial patterns, such as LPS [17]. LPS present on the outer membrane of Gram-negative bacteria is an endotoxin that can cause septic shock in animals [8,19]. LPS acts as a toxin by over-stimulating the innate immune signaling of the Toll-like receptor 4 (TLR4), which induces an exacerbated inflammatory response [8,19]. Nevertheless, IALP is able to detoxify bacterial LPS by dephosphorylation [16]. Lipid A is responsible for the toxicity of LPS and contains two phosphate groups coupled to glucosamines, allowing LPS to bind TLR4, triggering an inflammatory response (e.g., the release of pro-inflammatory cytokines) (Figure 1) [16]. However, the removal of one of the phosphate groups from lipid A by IALP generates a monophosphoryl lipid A, which is 100 times less toxic than unmodified lipid A [20,21,22]. Thus, IALP manages to prevent inflammatory processes that would be triggered if LPS were not dephosphorylated. The prevention of this inflammatory process may contribute to the maintenance of intestinal barrier integrity.

Several studies have been conducted with the intention of verifying the role of IALP in the defense of the intestinal mucosa and its interaction with the intestinal microbiota. The authors found that IALP is involved in the maintenance of normal gut microbial homeostasis and may have a therapeutic potential in the prevention/treatment of dysbiosis and infections caused by pathogenic bacteria [23].

In a study conducted in rats, Buchet et al. found that oral IALP supplementation favors the growth of commensal bacteria, increasing the restoration of the intestinal microbiota lost due to antibiotic treatment and inhibiting the growth of a pathogenic bacterium (*Salmonella typhimurium*) [16]. This is due to the ability of IALP to dephosphorylate other luminal phosphates such as adenosine triphosphate (ATP), increasing the secretion of bicarbonate ions that are important to control luminal pH and, consequently, inhibiting pathogens growth [8,24,25,26]. Thus, oral administration of IALP may also have therapeutic potential against dysbiosis and pathogenic infections [8,16]. In addition, an in vitro study by Shin et al. showed that IALP alleviated the toxicity of LPS in intestinal epithelial cells (IECs), inhibited the activity of nuclear factor kappa B (NF-κB), and blocked the invasion and displacement of bacterial pathogens into the IECs, assisting in the preservation of the intestinal barrier integrity [27]. 

Moreover, the role of endogenous IALP in the maintenance of intestinal barrier function has also been associated with the regulation of tight junction proteins (TJP) expression. In mouse embryonic fibroblasts (MEFs) generated from IALP-knockout mice, the protein expression levels of zolunin-1 (ZO-1), zonulin-2 (ZO-2), and occludin were significantly lower compared to the levels of these proteins in wild-type control cells [28]. In addition, overexpression of IALP in human colorectal adenocarcinoma Caco-2 and T84 cells markedly enhanced ZO-1 and ZO-2 expression [28]. These authors have further demonstrated that exogenous IALP pretreatment of Caco-2 cells is able to prevent the LPS-induced alteration in the location and assembly of TJP and ameliorate its effect on intestinal permeability [28]. 

Lastly, Larrick et al. reported decreased IALP activity with aging, along with decreased integrity of the intestinal epithelial barrier [29]. In addition, the leaky-gut-associated inflammation was prevented by supplementation with IALP, suggesting that IALP can be a major regulator of intestinal permeability. Therefore, it can be concluded that IALP plays a fundamental role in maintaining the intestinal barrier function and that it could be involved in different intestinal and metabolic diseases in which intestinal permeability is increased. 

## 3. IALP and Inflammatory Bowel Disease

Inflammatory bowel disease (IBD) comprises a group of disorders that involves chronic inflammation, including Crohn’s disease (CD) and ulcerative colitis (UC) [30]. Dysbiosis is a common feature in patients with IBD [31]. In addition, it has been shown that patients with CD or UC have reduced IALP mRNA expression and activity in intestinal biopsies compared to healthy individuals [32,33]. Moreover, Tuin et al. have shown that in these patients, IALP mRNA expression is reduced in inflamed intestinal tissue compared to non-inflamed intestinal tissue [32]. 

In animal models of experimental colitis triggered by dextran sulfate sodium (DSS) or piroxicam, oral or intrarectal administration of IALP has been shown to significantly reduce intestinal inflammation [32,34,35,36]. Results from these studies demonstrated that, in experimental colitis, a lower endogenous IALP activity is associated with higher severity of the intestinal injury, whereas intestinal inflammation can be reversed by supplementation with exogenous IALP. 

Danielak et al. studied the effect of IALP combined with moderate physical activity (voluntary wheel running) on experimental colitis induced by 2,4,6-trinitrobenzenesulfonic acid (TNBS) in mice fed a standard diet (SD) or high-fat diet (HFD) [37]. The authors found that in sedentary SD-fed mice, macroscopic and microscopic colitis was accompanied by a significant decrease in colonic blood flow and a significant increase in the colonic expression of tumor necrosis factor-alpha (TNF-α), IL-6, IL-1β, and leptin [37]. The same effects were exacerbated in sedentary HFD mice but reduced in active (exercising) animals, potentiated by IALP treatment, namely in obese mice [37]. They concluded that the combination of voluntary exercise and oral treatment with IALP synergistically favored healing of intestinal inflammation, strengthened antioxidant defense, and improved the course of experimental colitis [37]. In a subsequent study, the authors evaluated whether these effects were mediated by the intestinal microbiota [38]. They found that TNBS-induced colitis was worsened in obese sedentary mice and that IALP supplementation in combination with moderate physical activity attenuated the severity of murine colitis through a mechanism that involved the negative regulation of the intestinal cytokine/chemokine network and oxidative stress, an improvement of muscle strength and the modulation of the intestinal microbiota [38]. In particular, the authors proposed that the increase in the *Ruminococcus* genus, a butyrate-producing genus that has been found to exert anti-inflammatory properties, restoring and maintaining normal gastrointestinal tract function and integrity, may explain the effects observed [38]. 

In a study carried out on patients with UC, IALP was administered daily for 7 days via a duodenal catheter [39]. The authors found a short-term improvement in disease activity scores, accompanied by reductions in C-reactive protein and fecal calprotectin, within 21 days. Treatment with exogenous IALP was well tolerated and nonimmunogenic [39].

Since IALP can dephosphorylate and detoxify LPS, it is conceivable that IALP may exert these protective and anti-inflammatory effects via LPS detoxification. Thus, IALP may play an important role in IBD management, controlling LPS dephosphorylation and intestinal inflammation, and may be therapeutically effective, without the harmful risks associated with currently accepted therapies [40]. Furthermore, there is also evidence that intestinal permeability can play a fundamental role in the pathophysiology of IBD [41,42,43,44]. Thus, in addition to reducing inflammation, IALP may also contribute to IBD management by decreasing intestinal permeability, as explained in Section 2.

## 4. IALP and Necrotizing Enterocolitis (NEC)

Necrotizing enterocolitis (NEC) is the most common gastrointestinal emergency in premature infants, having an associated high mortality rate [45,46] and long-term associated morbidities, such as short bowel syndrome, nutritional deficiency, and delayed neurological development [46,47,48].

Newborn infants, especially those with very low birth weight, are more susceptible to sepsis due to prolonged hospitalizations, invasive instrumentation, underdeveloped innate immunity, and altered immune responses. These last two physiological states, together with an immature intestinal barrier, may contribute to NEC development [49,50].

Interestingly, IALP activity measured in fecal samples is significantly lower in infants with severe NEC compared to infants without NEC [46]. Since there is a decrease in IALP activity, further studies are needed to evaluate whether IALP supplementation could be effective in NEC prevention and treatment.

In 2014, a group of researchers investigated whether IALP supplementation could be protective of the premature intestine [51]. They evaluated premature newborn rats that were formula-fed with or without IALP supplementation. The authors showed that although there were no differences between groups regarding IALP activity, the animals supplemented with IALP had decreased mRNA expression of inflammatory cytokines in the terminal ileum [51]. IALP supplementation also decreased intestinal permeability (evaluated ex vivo) and the expression of inflammatory cytokines after exposure to LPS when compared to control animals fed with formula only [51]. These results support that IALP is beneficial for the premature intestine, reducing intestinal damage and inflammation caused by LPS [51].

Recent findings indicate that NEC is also preceded and accompanied by changes in the intestinal microbiota composition, which are associated with the host’s immune pathways responsible for activating intestinal inflammation [50,52]. By neutralizing LPS, IALP may prevent a cascade of pro-inflammatory signals in the intestine and contribute to the beneficial maturation of the microbiota [46]. This could constitute another mechanism by which IALP may be protective against NEC. 

Nevertheless, IALP supplementation is not the only way to prevent intestinal damage and inflammation that characterizes NEC. Preterm infants who receive human milk instead of formula have a lower incidence of NEC [53]. Additionally, as recently demonstrated by our group, premature infants who received human milk had their endogenous IALP activity increased [53]. In our recent work, very premature infants fed with mothers’ own milk (MOM) or donor human milk (DHM) had increased fecal ALP activity on the 26th day of life compared to formula-fed infants [53]. MOM and DHM concomitantly stimulated the growth of Bifidobacterium, although the mechanisms by which human milk consumption increase IALP activity remain to be further elucidated.

Lastly, as proposed by other authors, as the activity of the IALP precedes the beginning of the signaling cascades that trigger inflammation, the abundance and enzymatic activity of the IALP eliminated in the feces could be used as a specific biomarker in establishing the diagnosis of severe NEC, monitoring disease progression and surveilling high-risk infant groups [46].

## 5. IALP and Metabolic Dysfunction 

Metabolic syndrome is a set of several interrelated factors such as central obesity, hypertension, insulin resistance, and dyslipidemia that increase the risk of cardiovascular disease and type 2 diabetes [54,55]. 

Obesity is characterized by a state of low-grade chronic inflammation that could be a result of LPS and other microbial metabolites being absorbed along with dietary fats [56]. Evidence shows that high-fat diets decrease gut microbiota diversity (causing dysbiosis) and increase plasma concentration of LPS which can, in turn, trigger weight gain, inflammation, and insulin resistance [56,57].

Narisawa et al. have shown that IALP-deficient mice maintained on a high-fat diet showed faster body weight gain than wild-type animals [58]. Histological examination revealed accelerated transport of fat droplets through the intestinal epithelium and elevated serum triacylglyceride levels in IALP-deficient mice compared to wild-type mice, enlightening that IALP participates in the rate-limiting step that regulates fat absorption [58,59]. Nevertheless, although IALP-deficient mice might have gained more weight due to higher dietary fat absorption, the contribution of LPS circulating levels cannot be ruled out. In fact, these mice may have increased intestinal permeability (due to the lack of IALP) and, consequently, increased LPS levels (metabolic endotoxemia), which could have contributed to the increased weight gain [56].

In this regard, Kaliannan et al. have later confirmed that IALP knockout mice have increased gut permeability and suffer from metabolic endotoxemia, presenting obesity and associated metabolic disorders [60]. In this study, the authors also found that both endogenous and exogenous IALP inhibit LPS absorption and that oral IALP supplementation is capable of preventing and reversing metabolic syndrome [60].

Furthermore, a study published in 2015 evaluated the levels of IALP activity in fecal samples of diabetic (with or without overweight) and healthy non-diabetic patients [61]. This study related fecal IALP deficiency to T2D and showed that a high level of IALP is protective against diabetes, regardless of obesity [61].

Early childhood exposure to antibiotics has been implicated in the pathogenesis of metabolic syndrome later in adulthood [62]. In this context, Economopoulos et al. have shown that the use of antibiotics concomitantly with IALP supplementation early in life may have a preventive role against metabolic syndrome [62]. In this study, mice were treated for three intermittent 7-day cycles with azithromycin and supplemented with oral IALP. At the end of the last cycle, mice were treated with a regular diet for five weeks and then with a high-fat diet for another five weeks [62]. Co-administration of IALP with azithromycin prevented susceptibility to metabolic syndrome by decreasing total body weight, serum lipids, glucose levels, and liver lipids to the levels of control mice [62]. This effect may be explained through the prevention of antibiotic-induced changes in the microbiota by IALP.

In recent years, artificial sweeteners have been used as a substitute for sugar and as a weight control strategy [63]. However, several studies suggest that this exchange does not improve weight loss and may even contribute to the development of metabolic syndrome [64,65,66]. Recent evidence points out that artificial sweeteners, specifically aspartame (ASP), can have a direct effect on the intestinal microbiota that could explain the metabolic changes that occur after the consumption of high doses of artificial sweeteners [67,68,69]. In addition, a recently published cross-sectional study showed significant differences in the human gut microbiota when comparing the consumption of a diet rich in artificial sweeteners to that of a regular diet [70]. Furthermore, Frankenfeld et al. demonstrated a different bacterial composition in patients (healthy adults) after 4 days of consuming ASP, also showing that the consumption of low doses of ASP alters the intestinal microbiota [70].

ASP is the most used artificial sweetener used and is also known to be metabolized by intestinal esterases and peptidases to L-aspartic acid, L-phenylalanine (PHE), and methanol [71]. Since the amino acid PHE is a well-known IALP inhibitor [72], it may hinder the IALP-mediated detoxification of LPS [71]. As verified by Gul et al. in an isolated intestinal loop model, IALP activity was significantly reduced by the presence of ASP [71]. These results confirm that ASP can reduce the endogenous IALP activity at the intestinal brush border and lumen [71]. 

Thus, as already demonstrated in mice, the ingestion of 60 mg of ASP per liter of water altered the microbiota composition, verifying an increase in the number of bacteria of the *Enterobacteriaceae* and *Clostridium leptum* family (strain associated with the microbiota of obese individuals) [67]. Thus, by altering the composition of the intestinal microbiota and compromising the action of IALP, interfering with the neutralization of bacterial toxins such as LPS, ASP can contribute to the development of metabolic syndrome.

## 6. IALP—How Can It Be Modulated? 

Diet appears to have an important impact on the modulation of IALP [73]. Fasting dramatically decreases IALP activity, while feeding retrieves it [8,74].

From a nutritional perspective, strategies that lead to a healthy lifestyle should be adopted, namely, the adoption of a Mediterranean dietary pattern [75]. As demonstrated by Ismael et al., the activity of fecal IALP positively correlates with gut microbiota diversity promoted by a Mediterranean diet [76].

The Mediterranean diet is a diet rich in fiber, which can explain its effects on IALP activity. Fiber stimulates the growth and/or activity of bacteria in the colon, promoting the production of short-chain fatty acids that increase IALP activity [77,78].

As verified by the study of Gibson et al. carried out in rats, different food sources of fiber (uncooked potato starch; cooked potato starch; precooked corn flour; whole bran; guar gum; wheat bran; methylcellulose; oat bran) increased IALP activity in the colon. Intestinal mechanical stimulation induced by fiber intake increases the proliferation of colonic epithelium and, consequently, increases the activity of IALP [73,79]. 

On the other hand, the Mediterranean diet is low in trans fatty acids and privileges the consumption of food sources rich in polyunsaturated and monounsaturated fats. A diet supplemented with fish oil or corn oil increases IALP activity [80]. Triglycerides increase IALP expression and activity in the intestinal lumen; that is, the amount of fat in the diet determines the amount of IALP secretion [81,82,83,84]. On the other hand, the Mediterranean diet, which is rich in unsaturated fatty acids, increases IALP activity [81,85,86]. Different types of unsaturated fatty acids can influence IALP activity [85]. For example, sunflower oil decreases IALP activity compared to olive oil due to changes in phospholipid composition in the apical membrane of intestinal epithelial cells [85].

Trans fatty acids decrease IALP activity at the intestinal brush border membranes when the amount of linoleic acid in the diet is low, with no effect in the presence of greater amounts of linoleic acid [87]. 

The activity of IALP in the intestine can also be differently modulated by the microbiota [73]. Okazaki et al. showed a significant positive correlation between colon IALP activity, Bifidobacterium, mucins, and butyrate [88]. Some studies indicate that the consumption of fermentable oligosaccharides such as fructooligosaccharides and galactooligosaccharides (raffinose), as well as lactulose, modulate the gut microbiota by increasing both Bifidobacterium and butyrate [88,89,90,91,92]. Interestingly, butyrate is known to increase IALP activity [53]. However, further studies are needed to confirm the relationship between the non-digestible oligosaccharides-induced increase in colonic ALP and colonic barrier function [88].

In the human intestine, the main butyrate-producing bacteria belong to the *Firmicutes phylum*, in particular, *Faecalibacterium prausnitzii* and *Clostridium leptum* of the *Ruminococcaceae* family and *Eubacterium rectale* and *Roseburia* spp. of the family *Lachnospiraceae* [93]. There are also bacteria that use sugar and/or lactate to produce butyrate, such as *Eubacterium hallii* and *Anaerostipes* spp. [93,94]. Nevertheless, butyrate-producing bacteria may be present in higher numbers in the human intestine because members of *Actinobacteria*, *Bacteroidetes*, *Fusobacteria*, *Proteobacteria*, *Spirochaetes*, and *Thermotogae* are potential butyrate producers according to the genes they express, including those encoding enzymes that synthesize butyrate, such as butyryl—CoA dehydrogenase, butyryl-CoA transferase, and/or butyrate kinase [93].

**Figure 2:**
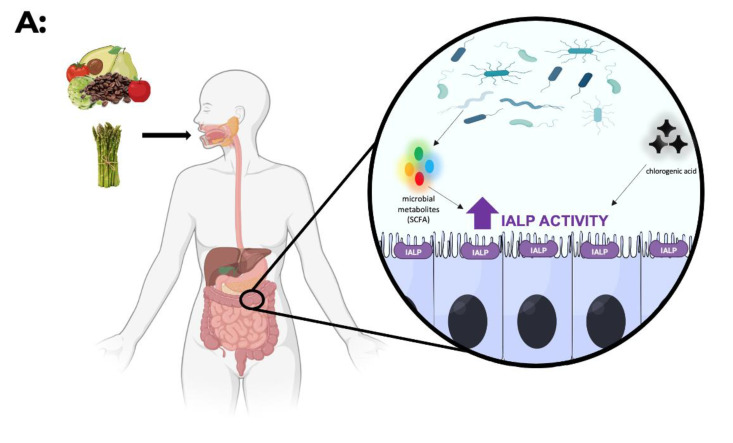

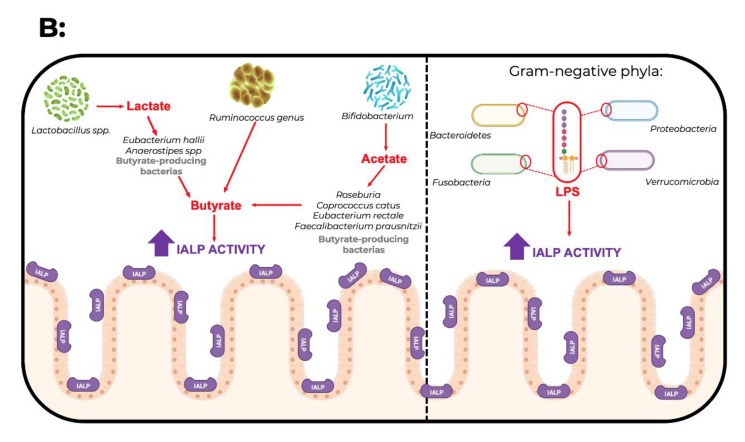
(**A**) Diet has an impact on the modulation of IALP (intestinal alkaline phosphatase) activity. Foods rich in fermentable fiber (e.g., artichoke, asparagus) are used as a substrate for the microbiota. This fermentation results in the production of short-chain fatty acids (e.g., butyrate) that increase IALP activity. On the other hand, consumption of foods rich in chlorogenic acid (coffee, artichoke, apple, pear, tomato, and avocado) also increases the activity/expression of IALP. (**B**) The gut microbiota may modulate the expression/activity of IALP. On the one hand, butyrate-producing bacteria (Gram-positive bacteria) may increase IALP due to the increase in butyrate. On the other hand, Gram-negative bacteria may also increase IALP due to the presence of LPS (the presence of LPS as a substrate may enhance enzyme activity).

In addition to butyrate, the production of other SCFAs is mediated by bacteria such as *Bifidobacterium* species (belonging to the *Phylum Actinobacteria*), which produce acetate and lactate during carbohydrate fermentation [93,94]. In addition, the mucin-degrading bacterium *Akkermansia muciniphila* (*Phylum Verrucomicrobia*) produces propionate and acetate [93,94]. In turn, acetate and lactate may be used as substrates by butyrate-producing bacteria (Figure 2B). 

A study carried out with pregnant women by Selma-Royo et al. did not observe a direct relationship between high-fat diets and IALP expression [95]. However, they found that ingesting fat (saturated (SFA) and monounsaturated (MUFA) fatty acids) increased *Firmicutes*, a strain associated with higher concentrations of IALP [95]. Furthermore, a lower abundance of the phylum *Proteobacteria* was associated with higher IALP activity [95]. 

Polyphenols seem to have an impact on the modulation of alkaline phosphatase activity; however, further work on this topic is needed since conclusions are scarce [96,97].

As verified by Zhou et al., chlorogenic acid showed a protective effect in the intestine of endotoxin-infused female Sprague-Dawley rats. With intragastric administration of 60 mg/kg of body weight of chlorogenic acid twice daily for 28 days, there was an improvement in endotoxin-induced intestinal damage and an increase in alkaline phosphatase activity. Thus, chlorogenic acid present in coffee and in a variety of vegetables and fruits may have an impact against increased intestinal permeability (Figure 2A) [97].

When used as a drug, IALP is usually administered by intravenous injections so that degradation and digestion in the stomach and upper intestinal tract does not occur [8]. Nevertheless, enteral administration of exogenous ALP has two main effects: detoxifying PAMPs and stimulating the production of endogenous IALP by the enterocyte. The mechanism by which exogenous IALP stimulates the production of endogenous IALP by the enterocyte is unknown, but it may be indirect, resulting from the reduction of local inflammation [98,99]. IALP administered intraperitoneally or intravenously contributes to the detoxification of circulating PAMPs without a positive feedback effect on IALP in the small intestine [98,99]. 

Regarding rectal enemas that allow the direct entry of IALP into the large intestine and exogenous IALP in the form of enteric-coated or delayed-release capsules, their effects have not yet been reported in human studies. 

There are some published studies on the administration of IALP in an animal model (Table 1) and in humans (Table 2); however, the conclusions are still scarce. Therefore, it is necessary to study and understand the effect of IALP supplementation and IALP modulation through the diet and microbiota in the prevention and treatment of diseases where its activity can be compromised, such as inflammatory bowel disease, necrotizing enterocolitis, and metabolic syndrome.

## 7. Concluding Remarks and Future Perspectives

To assess intestinal permeability in a research setting, the lactulose/mannitol test is usually used as a gold standard [104]. This test can aid in the non-invasive diagnosis of barrier dysfunction in patients with diarrheal diseases, malnutrition, surgical stress, Crohn’s disease, active inflammatory bowel disease, and irritable bowel syndrome, among other pathologies [105,106,107,108]. Nevertheless, it may not be feasible to implement routinely in clinical practice since urine is collected from 5 to 6 h after the ingestion of the solution containing lactulose and mannitol, making patients wait a long time for the results [104].

Other potential biomarkers such as fecal calprotectin and zonulin, as well as plasma intestinal fatty acid-binding protein (I-FABP), lipopolysaccharide-binding protein (LBP), and zonulin, have been proposed to assess intestinal permeability [109]. Nevertheless, fecal IALP activity would constitute a more promising alternative since it can be performed routinely by any clinical analysis laboratory, using a non-invasive single fecal sample collection, which translates into lower costs and fast results. Even so, further studies are necessary to validate the use of fecal IALP as a marker of intestinal permeability. 

## Figures and Tables

**Table 1:** Summary of the most recent studies on the administration of IALP in animal models.

Aim of Study	Route of Administration	Treatment Effect	Animal Model	Ref.
Examine whether co-administration of IALP with antibiotics early in life have a preventive role against metabolic syndrome	Oral IALP (100 units/mL drinking water) supplementation ad libitumfor three intermittent 7-day cycles	Co-administration of IALP with AZT early in life prevents mice from susceptibility to the later development of HFD-induced obesity and MetS	C57BL/6 mice	Economopoulos et al. (2016) [62]
Investigate whether oral IALP supplementation protects against alcohol-induced liver disease	Oral IALP supplementation (200 U/mL) for 10 days	IALP treatment protected mice from alcohol-induced hepatotoxicity and steatosis	Female C57BL/6 mice	Hamarneh et al. (2017) [100]
Evaluate whether the protective effect of IALP on DSS-induced colitis is mediated by the Toll-like receptor 4 (TLR4)/nuclear factor-kappa B (NF-κB) pathway	IALP (300 IU/day) via oral gavage for 7 days	Oral gavage administration of IALP significantly attenuated the severity of colitis via the TLR4/NF-κB pathway	WT C57BL/6 mice and TLR4^-/-^ mice	Hwang et al. (2018) [101]
Determine if bovine intestinal ALP (BiAP) infusion prevents AKI	BiAP was administered by continuous infusion (25 U/kg/hr) via a femoral central venous catheter	BiAP infusion corrects serum and tissue ALP deficiency and may prevent AKI	Porcine model of early infant CPB/DHCA -induced AKI	Davidson et al. (2019) [102]
Evaluate the effect of IALP combined with moderate physical activity (voluntary wheel running) on the experimental colitis	IALP (200 U/day) was administered intragastrical for 12 weeks	Oral IALP treatment synergistically favored healing of intestinal inflammation, strengthened the antioxidant defense, and ameliorated the course of experimental colitis	SD- and HFD-fed C57BL/6 mice with experimental colitis induced by TNBS	Danielak et al. (2021) [37]
Evaluate the effect of IALP combined with moderate physical activity (voluntary wheel running) on experimental colitis	Oral IALP supplementation (200 U/day) in drinking water for 2 weeks	Administration of IALP combined with moderate physical activity significantly reduced gross and microscopic inflammatory response and oxidative stress markers	HFD female C57BL/6J mice with experimental colitis induced by TNBS	Wojcik-Grzybek Dagmara et al. (2022) [38]

BiALP—bovine intestinal alkaline phosphatase; IALP—intestinal alkaline phosphatase; AZT—azithromycin; DSS—dextran sulfate sodium; CPB—cardiopulmonary bypass; DHCA—deep hypothermic circulatory arrest); SD—standard diet; MetS—metabolic syndrome; TLR4—Toll-like receptor 4; NF-κB—nuclear factor-kappa B; HFD—high-fat diet; SW—spinning wheel; TNBS—2,4,6-trinitrobenzenesulfonic acid; AKI—acute kidney injury.

**Table 2:** Studies on administration of alkaline phosphatase in humans.

Aim of Study	Study Design	Route of Administration	Treatment Effect	Sample Size/ Estimated Enrollment	Ref.
Evaluate the safety and preliminary efficacy of exogenous ALP administered to patients with UC	Interventionalallocation: N/A; intervention model: single group assignment; masking: none (open label); primary purpose: treatment	bIAP bolus 30,000 U/24 h for 7 consecutive days via a duodenal catheter	AP enzyme treatment was well tolerated and nonimmunogenic	21	M. Lukas et al. [39] (2010)
Evaluate the safety, pharmacokinetics, and pharmacodynamics of IV administration of exogenous ALP	Randomized; double-blind; placebo-controlled sequential protocols	Administered exogenous, 10 min IV infusions (three ascending doses) or 24–72 h continuous (132.5–200 U kg^−1^ 24 h^−1^) IV	Exogenous AP administration in severe sepsis patients may play a renal protective role	103	P. Pickkers et al. [103] (2009)
Evaluate whether alkaline phosphatase injections can reduce acute inflammation in patients with rheumatoid arthritis	Interventional (clinical trial); non-randomized	s.c. injections of bovine intestinal Alkaline Phosphatase daily subcutaneous treatment with two injections of 2000 IU bIAP for three days	Ongoing study	6	NCT01416493 *
Evaluate the efficacy and safety of bovine intestinal alkaline phosphatase (bIAP) in reducing the pro-inflammatory post-surgical responses	Allocation: randomized; intervention model: parallel assignment; masking: quadruple (participant, care provider, investigator, outcomes assessor); primary purpose: prevention	bIAP bolus and 8 h infusion intravenous as a bolus of bIAP (1000 IU) just prior to surgery followed by a 40 IU/kg bIAP infusion during the first 8 h post-surgery	Ongoing study	53	NCT01144611 *

* Clinical studies without published final results obtained from clinictrials.gov; bIAP—bovine intestinal alkaline phosphatase; s.c. —subcutaneous; UC—ulcerative colitis; IV—intravenous.

## Data Availability

Not applicable.
